# The mediating role of psychological entitlement in the relationship between relative deprivation and prosocial behavior among college students: a random intercept cross-lagged panel model

**DOI:** 10.3389/fpubh.2026.1821940

**Published:** 2026-06-03

**Authors:** Yihong Wang, Baojuan Ye, Jing Xu, Qi Dai

**Affiliations:** 1 School of Education, Jiangxi Normal University, Nanchang, China; 2 Centre for Psychological Education, Jiangxi Normal University, Nanchang, China; 3 Center of Mental Health Education and Research, School of Psychology, Jiangxi Normal University, Nanchang, China; 4 School of Physics and Optoelectronic Engineering, Shandong University of Technology, Zibo, Shandong, China

**Keywords:** college students, prosocial behavior, psychological entitlement, random intercept cross-lagged panel model, relative deprivation

## Abstract

**Background:**

College is a critical transitional period for students entering society, and prosocial behavior is crucial to their identity construction and mental health. Relative deprivation has become increasingly prevalent among college students due to higher education and intense employment competition. Although prior research has found a significant negative association between relative deprivation and prosocial behavior, their longitudinal relationship and underlying mechanism remain unclear. Therefore, this longitudinal study examined the mediating role of psychological entitlement in the relationship between relative deprivation and prosocial behavior among college students.

**Methods:**

This study used the Random Intercept Cross-Lagged Panel Model (RI-CLPM) with three waves of longitudinal data. Participants comprised 1,628 college students aged 16–25 years (*M* = 19.69, *SD* = 1.36). They completed a paper questionnaire that assessed their levels of relative deprivation, psychological entitlement, and prosocial behavior.

**Results:**

Results revealed that relative deprivation was significantly and positively associated with psychological entitlement at the between-person level. Furthermore, both were significantly and negatively associated with prosocial behavior. At the within-person level, prosocial behavior negatively predicted longitudinal relative deprivation, whereas relative deprivation did not predict longitudinal prosocial behavior. Furthermore, psychological entitlement mediated the longitudinal relationship between relative deprivation and prosocial behavior.

**Conclusion:**

These findings emphasize the dynamic interaction between relative deprivation, psychological entitlement, and prosocial behavior, and highlight the mediating role of psychological entitlement. They may offer meaningful insights into prosocial behavior, psychological entitlement, and relative deprivation among college students.

## 1 Introduction

Prosocial behavior refers to individuals' voluntary actions performed by individuals to improve others' wellbeing or support group interests (1, 2). According to the latest data, the Chinese college student population has reached nearly 39 million (3). Prosocial behavior is an essential indicator of social maturity among college students. It improves the quality of interpersonal relationships, enhances social adaptation skills, and promotes mental health (4). Furthermore, it helps shape positive values and develop healthy personalities, essential for students' career development and social engagement (2, 4). Therefore, understanding the mechanisms behind prosocial behavior in college students is an important research area.

As higher education expands and the post-pandemic job market recovers gradually, college students are confronted with growing academic and employment pressures (5). Moreover, relative deprivation is becoming increasing prevalent among this group (6). Numerous studies have reported that relative deprivation is negatively associated with prosocial behavior in college students (7–9). Consequently, reduction of relative deprivation and promotion of prosocial behavior are essential components of mental health education for college students (10). Relative deprivation is considered a predictor of prosocial behavior; however, an inverse path is also possible. Additionally, the mechanism underlying the longitudinal relationship between relative deprivation and prosocial behavior remains unclear. Therefore, this study adopted a longitudinal design to explore the potential bidirectional association between relative deprivation and prosocial behavior and investigate its underlying mediating mechanism. The findings may offer empirical insights into factors associated with relative deprivation and prosocial behavior among college students.

### 1.1 Reciprocal association between relative deprivation and prosocial behavior

#### 1.1.1 Relative deprivation as a possible antecedent for prosocial behavior

Relative deprivation refers to the subjective cognitive and emotional state of disadvantage that individuals experience when comparing themselves to a reference group, often accompanied by anger and dissatisfaction (11, 12). College students, who may have unstable emotional development, are particularly susceptible to social comparison within campus hierarchies and peer groups (11). Increasing competition in academia and employment can further intensify these comparative tendencies, which may lead to feelings of relative deprivation (5). Research has revealed that individuals experiencing relative deprivation are more likely to reject prosocial behavior; furthermore, higher relative deprivation is associated with lower prosocial behavior (7–9). Guo et al.'s (13) longitudinal study reported that college students who consistently felt deprived over time had stronger motives for revenge, which made them more likely to engage in cyberbullying. Prosocial behavior often entails personal costs, including time, energy, money, or other valuable resources (14). Therefore, individuals assess their circumstances and capabilities before engaging in such behavior, and this evaluation ultimately influences their decision (7, 15). Relative deprivation is defined as an individual's perception that they are deprived of outcomes or benefits they believe they deserve, resulting in a relatively disadvantaged position and triggering feelings of loss or victimization (12). According to the conservation of resources theory, people focus on protecting and acquiring resources rather than helping others when their resources are perceived as inadequate or threatened; furthermore, this tendency is associated with lower levels of prosocial behavior (8, 16). Therefore, we hypothesized that relative deprivation may be negatively related to later prosocial behavior across time among college students.

#### 1.1.2 Prosocial behavior as a possible antecedent for relative deprivation

Although relative deprivation is generally expected to be correlated with prosocial behavior, the reverse effect is also possible. There are some reasons to believe that prosocial behavior may be negatively associated with subsequent relative deprivation over time. Engaging in prosocial behavior can evoke positive emotions, such as empathy, joy, and a sense of achievement (17, 18). The broaden-and-build theory of positive emotions suggests that positive emotions can expand an individual's cognitive perspective and help alleviate the attentional constraints caused by negative emotions often associated with relative deprivation, such as anger and jealousy. Furthermore, they may reduce an individual's focus on personal gains and losses, and ultimately lower the impact of relative deprivation among college students (12, 19). Indirect evidence also supports our hypotheses. Researchers have reported that engaging in prosocial behavior can effectively satisfy individuals' fundamental psychological needs: competence, autonomy, and relatedness (20). According to the self-determination theory, these basic psychological needs are crucial for individuals to develop a stable internal value system. This, in turn, reduces the tendency to seek self-worth through external social comparisons, which is linked to lower levels of relative deprivation (10, 21). Gao et al. (22) discovered that individuals' passive exposure to green spaces can help alleviate feelings of relative deprivation by satisfying basic psychological needs. Similarly, Xie et al. (23) identified fulfillment of these psychological needs as a protective factor against the fear of missing out among college students; furthermore, reduced relative deprivation mediated this effect. Therefore, we hypothesized that prosocial behavior may be negatively related to later relative deprivation across time among college students. This study aimed to further clarify the longitudinal bidirectional relationship between relative deprivation and prosocial behavior among college students.

### 1.2 The possible mediator of psychological entitlement

Psychological entitlement refers to a stable and pervasive belief that one deserves special treatment from others or society and is even exempt from common social obligations (24). Relative deprivation refers to an individual's perception of the gap between their actual resources and what they believe they should possess (12). This gap may stem from various factors, such as negative childhood experiences (25), insufficient rewards for their efforts (26), or lack of respect and recognition they feel they deserve (27). This subjectively perceived gap elicits a strong sense of unfairness (28, 29). According to the equity theory, individuals who perceive unfair losses tend to seek greater future benefits as compensation. They may even avoid social responsibilities after wrongdoing, which can strengthen their psychological entitlement to preferential treatment (26). Thus, relative deprivation can serve as an antecedent of psychological entitlement. Ding et al.'s cross-sectional study (29) found that relative deprivation was positively associated with psychological entitlement among college students. Furthermore, psychological entitlement could be regarded as an antecedent of prosocial behavior. Previous research has also indicated that psychological entitlement is a maladaptive subjective cognition often associated with negative outcomes (24, 30). According to the social information processing model, individuals' social behavior relies on a series of processing stages (31). Psychological entitlement, as a maladaptive cognitive bias, can foster egocentrism during information processing (30). This egocentrism reduces social morality, responsibility (32), and empathy (24), thereby promoting selfishness (26), immoral behavior (33), and aggression (24). Babu et al. (34) found that employees with high psychological entitlement tend to ignore others' interests and exhibit moral disengagement, which makes it difficult for them to internalize prosocial values. Consequently, they exhibit significantly lower prosocial motivation than those with low psychological entitlement. Additionally, Zhang and Qin (35) examined green consumption behavior and discovered that psychological entitlement significantly reduces prosocial behavior. However, existing research has predominantly examined the association between psychological entitlement and negative outcomes; its influence on positive behaviors remains underexplored, particularly among college students. Therefore, examining how psychological entitlement may predict subsequent prosocial behavior within this population is important. Evidence suggests that relative deprivation is positively correlated with psychological entitlement, which, in turn, negatively predicts prosocial behavior. Thus, psychological entitlement may function as a mediator in the longitudinal pathway between relative deprivation and prosocial behavior.

We also investigated the mediating role of psychological entitlement in the pathway from prosocial behavior to relative deprivation, as psychological entitlement may be predicted by prosocial behavior and subsequently predict relative deprivation over time. According to Adler's theory of social interest, engaging in prosocial behavior and contributing to others fosters social connectedness and leads individuals to view themselves as essential community members; furthermore, it enables them to achieve self-worth rather than merely pursuing self-serving needs (36, 37). By contrast, psychological entitlement reflects a self-centered tendency that prioritizes one's own needs and interests over that of others', which can lead individuals to believe they deserve special treatment (24). The cognitive framework of prosocial behavior directly challenges the beliefs of those who are psychologically entitled, reducing their focus on self-interest and their perception of deserving special treatment (18, 24). Hoffman and Wallach (37) found that students who participated in community gardening volunteer activities scored significantly lower on entitlement than those who did not. Furthermore, indirect evidence may help clarify the view that prosocial behavior reduces psychological entitlement. Zhou et al. (38) conducted a 35-day daily study and found that individuals who more frequently experienced positivity resonance had significantly lower levels of psychological entitlement. Lee et al. (39) conducted two longitudinal experiments and demonstrated that regularly recording grateful events significantly reduced individuals' psychological entitlement. Importantly, both gratitude and positivity resonance are closely linked to prosocial behavior (38, 40); these results provide indirect support for our hypothesis. Therefore, prosocial behavior may be negatively associated with subsequent psychological entitlement over time. Additionally, psychological entitlement can be regarded as an antecedent in shaping relative deprivation. The relative deprivation theory suggests that a sense of entitlement is essential for experiencing relative deprivation (12). Psychological entitlement raises individuals' expectations of what they believe they deserve, widens the perceived gap between their actual situation and what they feel entitled to, and intensifies their relative deprivation (30). Bernstein and Crosby (41) found that participants with higher entitlement reported a stronger sense of deservingness and had greater feelings of injustice and deprivation when they failed to obtain expected outcomes. Vatankhah and Raoofi (42) further demonstrated that flight attendants with higher psychological entitlement exhibited significantly stronger egoistic deprivation when encountering negative work outcomes. Additionally, indirect evidence supports the potential pathway from psychological entitlement to relative deprivation. Research has revealed that psychological entitlement is associated with various negative subjective experiences. Individuals with high levels of psychological entitlement are more likely to perceive unfairness (43) and report lower job satisfaction (44) and lower wellbeing (45). These negative experiences are closely related to relative deprivation (12, 46). Thus, psychological entitlement may be an antecedent in shaping relative deprivation. Evidence suggests that prosocial behavior may predict lower psychological entitlement, which, in turn, may predict lower relative deprivation. Thus, we hypothesized that psychological entitlement may mediate the longitudinal relationship between prosocial behavior and relative deprivation.

### 1.3 The current study

Although existing research has examined the relationships between relative deprivation, psychological entitlement, and prosocial behavior, longitudinal studies that simultaneously incorporate all three variables remain scarce. Consequently, understanding of their longitudinal associations remains limited. Drawing on the dynamic transactional model, which posits that variables influence each other reciprocally over time (47, 48), this study proposed that relative deprivation, psychological entitlement, and prosocial behavior would form complex, dynamic relationships. Moreover, the cross-lagged panel model (CLPM), widely used to examine such dynamic associations, has been criticized for failing to adequately differentiate between-person and within-person effects (49). Considering both stable, trait-like characteristics and dynamic fluctuations occurring within individuals over time is essential when examining the relationships between relative deprivation, psychological entitlement, and prosocial behavior. Conversely, the random intercept cross-lagged panel model (RI-CLPM) incorporates random intercepts to account for individuals' trait-like stable levels without assuming a linear growth pattern across time. This model estimates within-person reciprocal paths using residuals from repeated measurements, and can separate between-person stability from within-person dynamic changes (49). Thus, we used the RI-CLPM to (a) examine whether the main variables were related to one another at a trait level (between-person effect), (b) characterize changes in relative deprivation, psychological entitlement, and prosocial behavior across time, controlled for trait-like stability, and (c) determine whether the reciprocal link between relative deprivation and prosocial behavior would be mediated by psychological entitlement.

Moreover, understanding the importance of temporal precedence is essential for clarifying the temporal logic of longitudinal mediation (49, 50). Observational longitudinal studies should follow a basic methodological rule to advance beyond simple correlation and establish directional temporal associations; they should measure the predictor, mediator, and outcome in a clear time-ordered sequence. Specifically, the predictor should be assessed before the mediator, and the mediator should be evaluated before the outcome (51, 52). This three-wave structure ensures that earlier deviations in one variable can predict later changes in another, establishing a necessary sequential pattern, though not sufficient, for causal interpretation. By positioning psychological entitlement at T2, between T1 and T3, we met the temporal precedence requirement for both indirect paths. This arrangement allowed us to examine two within-person longitudinal indirect paths: (a) T1 relative deprivation predicts T2 psychological entitlement, which predicts T3 prosocial behavior, and (b) T1 prosocial behavior predicts T2 psychological entitlement, which subsequently predicts T3 relative deprivation. This temporal sequencing is not merely a statistical formality, it reflects the theoretical proposition that psychological entitlement occupies a structurally intermediate position (47). Furthermore, according to the dynamic transactional model, reciprocal interactions between individuals and contexts are often transmitted through intraindividual psychological and cognitive processes (47, 48). Relative deprivation and prosocial behavior may reflect an individual's subjective perception of their context and their behavioral response, respectively. Psychological entitlement may serve as the core cognitive mechanism such that it (a) correlates with perceived deprivation and subsequent changes in prosocial behavior and (b) varies with prosocial engagement and corresponds to fluctuations in subsequent deprivation perceptions. This structurally intermediate position suggests that psychological entitlement may mediate cross-temporal effects in both directions. Additionally, this framework aligns with recent empirical applications of the transactional model (47, 48). A recent study used a three-wave RI-CLPM to investigate how emotion dysregulation mediates the relationship between sleep disturbances and aggressive behavior (53) and positioned this cognitive-emotional variable at an intermediate time point to illustrate cross-temporal associations among the variables. This study adopted a similar analytical approach and placed psychological entitlement at T2 to facilitate its role as an important cognitive pathway linking relative deprivation and prosocial behavior over time.

Therefore, we hypothesized that:

Hypothesis 1 (H1): relative deprivation can predict changes in prosocial behavior over time.

Hypothesis 2 (H2): prosocial behavior can predict changes in relative deprivation over time.

Hypothesis 3 (H3): psychological entitlement can mediate the longitudinal effect of relative deprivation on prosocial behavior.

Hypothesis 4 (H4): psychological entitlement can mediate the longitudinal effect of prosocial behavior on relative deprivation.

## 2 Methods

### 2.1 Participants and procedure

This study recruited participants from a Chinese University via convenience sampling. The Human Research Ethics Committee of the School of Psychology at Jiangxi Normal University approved this study (IRB-JXNU-PSY-2025042). Data were collected at three time points with a three-month interval: January 2025 (T1), April 2025 (T2), and July 2025 (T3). A total of 1,799 questionnaires were initially obtained. After excluding invalid responses (e.g., careless or patterned answers), 1,628 valid cases were included in the final analysis. The final sample comprised 812 males (49.9%) and 816 females (50.1%), all participants aged 16–25 years (*M* = 19.69, *SD* = 1.36). Informed consent was obtained from all participants, and guardian consent was required for minor participants aged 16–17 years. By grade level, the sample included 585 freshmen (35.9%), 609 sophomores (37.4%), 170 juniors (10.4%), and 264 seniors (16.2%).

The gender (χ^2^ (1) = 0.75, *p* > 0.05), age (*t* = −0.26, *p* > 0.05), relative deprivation (*t* = −1.52, *p* > 0.05), psychological entitlement (*t* = −0.75, *p* > 0.05), and prosocial behavior (*t* = 0.74, *p* > 0.05) of the dropout group (*N* = 171) showed no significant differences in T1 test scores, indicating no systematic dropout in this study.

### 2.2 Measures

#### 2.2.1 Relative deprivation

Relative deprivation was measured by four items of the Relative Deprivation Questionnaire (54). The scale was developed in Chinese, and responses are rated on a 6-point scale ranging from 1 (strongly disagree) to 6 (strongly agree). The scale has demonstrated good reliability and validity in Chinese samples (55). An example question is, “Given all the effort and sacrifice I have put in, my life should be better than it is now”. Item mean scores were calculated from the responses, with higher scores indicating high relative deprivation. In this study, the Cronbach's α values were 0.83, 0.83, 0.85.

#### 2.2.2 Psychological entitlement

Psychological entitlement was measured by nine items Chinese Version (56) of the Psychological Entitlement Scale (24). Responses are rated on a 7-point scale, ranging from 1 (strongly disagree) to 7 (strongly agree). The scale has demonstrated good reliability and validity in Chinese samples (57). An example question is, “I really feel I deserve more praise than others”. Item mean scores were calculated from the responses, with higher scores indicating high psychological entitlement. In this study, the Cronbach's α values were 0.92, 0.92, 0.93.

#### 2.2.3 Prosocial behavior

Prosocial behavior was measured by the Chinese Version (58) of the prosocial behavior subscale of the Strengths and Difficulties Questionnaire (59). Responses are rated on a 3-point scale, ranging from 1 (strongly disagree) to 3(strongly agree). This scale has demonstrated good reliability and validity in Chinese samples (60). An example question is, “I often share things with others.” Item mean scores were calculated from the responses, with higher scores indicating high prosocial behavior. In this study, the Cronbach's α values were 0.88, 0.90, 0.90.

### 2.3 Statistical analysis

SPSS version 27.0 was used for preliminary analysis, Harman's one-factor test and confirmatory factor analysis (CFA, performed in Mplus 8.3), descriptive statistics, and Pearson's correlation analyses. Additionally, intraclass correlation coefficients (ICCs) were calculated via a one-way random-effects analysis of variance (ANOVA). Subsequent analyses were conducted using Mplus 8.3. Measurement invariance tests were conducted at three different time points. RI-CLPM was established to explore reciprocal relationships among the variables and to distinguish between within-person and between-person effects. The baseline model was set as a freely estimated model. Subsequently, constrained models were constructed by progressively equating autoregressive and cross-lagged paths across time. If model fit deterioration was insignificant, the more parsimonious constrained model was selected. Finally, the longitudinal mediating effect of psychological entitlement between relative deprivation and prosocial behavior was examined using 2,000 bootstrap samples. The mediating effect was considered statistically significant if the 95% confidence interval did not include zero (52).

Parameters of the RI-CLPM were estimated using maximum likelihood estimation (ML). To assess the temporal stability of longitudinal effects, four nested models were constructed sequentially. The unconstrained baseline model allowed all cross-wave autoregressive and cross-lagged paths to be freely estimated. The autoregressive constrained model restricted only autoregressive paths to be time-invariant. The cross-lagged constrained model restricted only cross-lagged paths to be time-invariant. The fully constrained model set autoregressive effects, bidirectional cross-lagged paths, and concurrent residual covariances as equivalent across waves. Within-person residual variances at each wave were estimated without cross-wave restrictions. Factor loadings of random intercepts were fixed at 1 for model identification purposes (50). The optimal parsimonious model was selected based on the criteria of Δcomparative fit index (CFI) ≤ 0.01, and Δroot mean square error of approximation (RMSEA) ≤ 0.015 (61).

## 3 Results

### 3.1 Common method bias assessment

Harman's single-factor test showed that three factors with eigenvalues greater than 1 emerged in three measurements (62). The first factor explained 38.98, 38.11, and 38.24% of the variance, respectively, and all were below the critical threshold of 40%. The CFA indicates that the one-factor model fits the data poorly [χ^2^/df = 21.23, CFI = 0.432, TLI = 0.410, and RMSEA = 0.111 (0.110, 0.113)], and factor loadings ranged from 0.254 to 0.958 and residual variances were substantial, suggest that no single factor explains the majority of the variance (63, 64). These suggest that common method bias is unlikely to have a large impact on our findings.

### 3.2 Descriptive statistics and correlation analyses

We first analyzed the bivariate relations among the variables (Table 1). Descriptive statistics and correlation analyses for the study variables at each time point are presented in Table 1. Intraclass correlation (ICC) analyses revealed ICC values of 0.593, 0.679, and 0.549 for relative deprivation, psychological entitlement, and prosocial behavior, respectively. These values indicate that 59.3, 67.9, and 54.9% of the variance was attributable to between-person differences, reflecting stable trait-like components across individuals.

**Table 1:** Descriptive statistics and correlations among variables.

Variable	Mean ±*SD*	1	2	3	4	5	6	7	8	9	10
1. Gender											
2. Age	19.69± 1.36	−0.31^**^									
3. Relative deprivation(T1)	4.06 ± 1.00	−0.02	0								
4. Relative deprivation(T2)	4.08 ± 0.98	−0.02	0.02	0.41^**^							
5. Relative deprivation(T3)	4.01 ± 1.04	0	0	0.24^**^	0.34^**^						
6. Psychological entitlement(T1)	4.48 ± 1.16	−0.02	−0.01	0.28^**^	0.29^**^	0.19^**^					
7. Psychological entitlement(T2)	4.53 ± 1.14	0.01	−002	0.38^**^	0.32^**^	0.26^**^	0.44^**^				
8. Psychological entitlement(T3)	4.33 ± 1.21	0	0	0.25^**^	0.31^**^	0.35^**^	0.33^**^	0.48^**^			
9. Prosocial behavior(T1)	1.69 ± 0.57	−0.01	−0.02	−0.33^**^	−0.35^**^	−0.22^**^	−0.40^**^	−0.36^**^	−0.26^**^		
10. Prosocial behavior(T2)	1.77 ± 0.60	0.04	−0.04	−0.21^**^	−0.32^**^	−0.27^**^	−0.36^**^	−0.34^**^	−0.31^**^	0.36^**^	
11. Prosocial behavior(T3)	1.77 ± 0.61	0	0.01	−0.20^**^	−0.22^**^	−0.28^**^	−0.15^**^	−0.31^**^	−0.27^**^	0.23^**^	0.28^**^

^**^P < 0.01.

### 3.3 Measurement invariance tests

Measurement invariance tests were conducted for the scales that assessed relative deprivation, psychological entitlement, and prosocial behavior across three waves. We used the nested model comparisons and sequentially assessed configural, metric, and scalar invariance. Subsequently, we compared model fit indices across various invariance constraints. Acknowledging the sensitivity of chi-square values to sample size, we utilized changes in CFI and RMSEA to assess significant shifts in model fit. Following the criteria established, test invariance across the three assessments was indicated when ΔCFI ≤ 0.01 and ΔRMSEA ≤ 0.015. Results revealed that the measurement invariance of the longitudinal data for each scale fit well and satisfied the criteria for scalar invariance (Table 2), which allowed for subsequent analyses.

**Table 2:** Measurement invariance tests.

Variable	Model	χ^2^	*df*	CFI	TLI	RMSEA	SRMR	Model Comparison	ΔCFI	ΔRMSEA
Relative deprivation	M0	94.109	39	0.993	0.988	0.029	0.016			
	M1	97.475	45	0.993	0.990	0.027	0.018	M1-M0	0	−0.002
	M2	113.069	65	0.992	0.991	0.021	0.021	M2-M1	−0.001	−0.006
Psychological entitlement	M0	389.795	294	0.996	0.996	0.014	0.015			
	M1	406.280	310	0.996	0.996	0.014	0.018	M1-M0	0	0
	M2	464.191	328	0.995	0.994	0.016	0.023	M2-M1	−0.001	0.002
Prosocial behavior	M0	133.958	72	0.995	0.993	0.023	0.013			
	M1	136.465	80	0.996	0.994	0.021	0.014	M1-M0	0.001	−0.002
	M2	187.904	90	0.993	0.991	0.026	0.029	M2-M1	−0.003	0.005

M0, configural invariance model.

M1, metric invariance model.

M2, scalar invariance model.

### 3.4 Random-intercept cross-lagged panel model analysis

After controlling for gender, the unconstrained RI-CLPM showed acceptable fit (Table 3). Considering a balance between parsimony and fit, Model M4 was selected as the final model (χ^2^*/df* = 1.81, CFI = 0.995, TLI = 0.988, RMSEA = 0.022, SRMR = 0.019).

**Table 3:** Random-intercept cross-lagged panel model (RI-CLPM) analysis.

Model	χ^2^	*df*	CFI	TLI	RMSEA	SRMR	Model Comparison	ΔCFI	ΔRMSEA
M1	11.044	9	0.999	0.997	0.012	0.009			
M2	23.976	12	0.996	0.986	0.025	0.017	M2–M1	−0.003	0.013
M3	20.934	15	0.998	0.994	0.016	0.015	M3–M1	−0.001	0.004
M4	32.493	18	0.995	0.988	0.022	0.019	M4–M1	−0.004	0.010

M1, unconstrained model.

M2, autoregressive paths fixed to be time-invariant.

M3, cross-lagged paths fixed to be time-invariant.

M4, autoregressive and cross-lagged paths fixed to be time-invariant.

#### 3.4.1 Between-person relations

**Figure 1:**
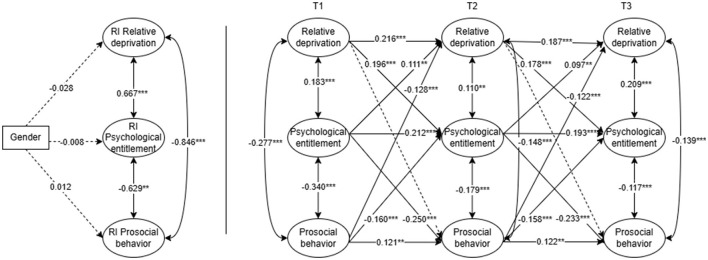
Random intercept cross-lagged panel models (RI-CLPMs) for relative deprivation, psychological entitlement, and prosocial behavior. **p < 0.01, ***p < 0.001.

A significant correlation was observed between the random intercepts of relative deprivation, psychological entitlement, and prosocial behavior at the between-person level. Specifically, relative deprivation was significantly positively associated with psychological entitlement. Furthermore, both relative deprivation and psychological entitlement were significantly negatively associated with prosocial behavior. These results indicated that college students who experienced higher levels of relative deprivation also reported higher levels of psychological entitlement. Furthermore, college students who experienced higher levels of both relative deprivation and psychological entitlement reported fewer prosocial behaviors (Table 4 and Figure 1).

**Table 4:** RI-CLPM estimates (full standardized).

	Std. Est	S.E.	*P*	95%CI
Covariate				
Gender → relative deprivation	−0.028	0.057	0.619	[−0.121, 0.065]
Gender → psychological entitlement	−0.008	0.045	0.862	[−0.082, 0.066]
Gender → prosocial behavior	0.012	0.050	0.816	[−0.071, 0.094]
Between-person effects				
Relative deprivation with psychological entitlement	0.667	0.189	< 0.001	[0.356, 0.977]
Relative deprivation with prosocial behavior	−0.846	0.218	< 0.001	[−1.205, −0.487]
Psychological entitlement with prosocial behavior	−0.629	0.181	0.001	[−0.927, −0.331]
Within-person effects				
Autoregressive paths				
Relative deprivation_T1_ → relative deprivation_T2_	0.216	0.043	< 0.001	[0.145, 0.287]
Relative deprivation_T2_ → relative deprivation_T3_	0.187	0.039	< 0.001	[0.123, 0.252]
Psychological entitlement_T1_ → psychological entitlement_T2_	0.212	0.047	< 0.001	[0.135, 0.289]
Psychological entitlement_T2_ → psychological entitlement_T3_	0.193	0.049	< 0.001	[0.113, 0.273]
Prosocial behavior_T1_ → prosocial behavior_T2_	0.121	0.035	0.001	[0.063, 0.180]
Prosocial behavior_T2_ → prosocial behavior_T3_	0.122	0.035	0.001	[0.064, 0.181]
Cross-lagged paths				
Relative deprivation_T1_ → psychological entitlement_T2_	0.196	0.036	< 0.001	[0.138, 0.255]
Relative deprivation_T1_ → prosocial behavior_T2_	−0.016	0.031	0.590	[−0.067, 0.034]
Relative deprivation_T2_ → psychological entitlement_T3_	0.178	0.032	< 0.001	[0.124, 0.231]
Relative deprivation_T2_ → prosocial behavior_T3_	−0.015	0.028	0.591	[−0.062, 0.031]
Psychological entitlement_T1_ → relative deprivation_T2_	0.111	0.034	0.001	[0.056, 0.167]
Psychological entitlement_T1_ → prosocial behavior_T2_	−0.25	0.032	< 0.001	[−0.304, −0.197]
Psychological entitlement_T2_ → relative deprivation_T3_	0.097	0.031	0.002	[0.047, 0.148]
Psychological entitlement_T2_ → prosocial behavior_T3_	−0.233	0.032	< 0.001	[−0.286, −0.180]
Prosocial behavior_T1_ → relative deprivation_T2_	−0.128	0.030	< 0.001	[−0.178, −0.079]
Prosocial behavior_T1_ → psychological entitlement_T2_	−0.160	0.031	< 0.001	[−0.211, −0.109]
Prosocial behavior_T2_ → relative deprivation_T3_	−0.122	0.028	< 0.001	[−0.168, −0.075]
Prosocial behavior_T2_ → psychological entitlement_T3_	−0.158	0.029	< 0.001	[−0.206, −0.109]
Residuals				
Relative deprivation_T1_ with psychological entitlement_T1_	0.183	0.052	< 0.001	[0.097, 0.269]
Relative deprivation_T2_ with psychological entitlement_T2_	0.110	0.040	0.007	[0.044, 0.177]
Relative deprivation_T3_ with psychological entitlement_T3_	0.209	0.032	< 0.001	[0.156, 0.261]
Relative deprivation_T1_ with prosocial behavior_T1_	−0.227	0.048	< 0.001	[−0.307, −0.148]
Relative deprivation_T2_ with prosocial behavior_T2_	−0.148	0.035	< 0.001	[−0.205, −0.091]
Relative deprivation_T3_ with prosocial behavior_T3_	−0.139	0.028	< 0.001	[−0.186, −0.092]
Psychological entitlement_T1_ with prosocial behavior_T1_	−0.34	0.043	< 0.001	[−0.410, −0.270]
Psychological entitlement_T2_ with prosocial behavior_T2_	−0.179	0.036	< 0.001	[−0.237, −0.120]
Psychological entitlement_T3_ with prosocial behavior_T3_	−0.117	0.033	< 0.001	[−0.171, −0.063]

#### 3.4.2 Within-person relations

At the within-person level (Table 4 and Figure 1), all auto-regressive paths in the cross-lagged models were significant, as were the bidirectional correlation paths within the same time points. Subsequently, the cross-lagged paths revealed that changes in relative deprivation from T1 to T2 positively predicted changes in psychological entitlement at T2 and T3. However, they did not significantly predict changes in prosocial behavior at T2 and T3. Meanwhile, changes in psychological entitlement from T1 to T2 positively and negatively predicted changes in relative deprivation and prosocial behavior, respectively, at T2 and T3. Additionally, changes in prosocial behavior from T1 to T2 negatively predicted changes in relative deprivation and psychological entitlement at T2 and T3. Finally, mediation analysis revealed that psychological entitlement (T2) mediated the effect of relative deprivation (T1) on prosocial behavior (T3) [β_*indirect*_ = −0.046, *p*<*0.001*, 95% CI (−0.064, −0.027)]. Additionally, psychological entitlement (T2) mediated the effect of prosocial behavior (T1) on relative deprivation (T3) [β_*indirect*_ = −0.016*, p*<*0.01*, 95% CI (−0.025, −0.006)].

## 4 Discussion

This study used the RI-CLPM to investigate the mediating role of psychological entitlement at within-person level between relative deprivation and prosocial behavior. We analyzed three waves of longitudinal data collected from Chinese college students. Overall, the results partially support our hypothesis, controlled for gender. Relative deprivation did not negatively predict subsequent prosocial behavior; thus, H1 was not supported. However, prosocial behavior negatively predicted subsequent relative deprivation. Thus, H2 was supported. Additionally, psychological entitlement mediated the bidirectional relationship between relative deprivation and prosocial behavior. Thus, H3 and H4 were supported. These results provide insights into the longitudinal associations between relative deprivation, psychological entitlement, and prosocial behavior among college students at both the within-and between-person levels.

### 4.1 Between-person results

A significant correlation was observed between the random intercepts of relative deprivation, psychological entitlement, and prosocial behavior at the between-person level. Specifically, relative deprivation was significantly positively associated with psychological entitlement. Furthermore, both relative deprivation and psychological entitlement were significantly negatively associated with prosocial behavior (7, 8, 20, 22). These findings are consistent with those of previous research that report a negative relationship between relative deprivation and prosocial behavior, positive relationship between relative deprivation and psychological entitlement (29, 42), and negative relationship between psychological entitlement and prosocial behavior (35, 37). This study offers new empirical evidence regarding their relationships between college students at the between-person level.

### 4.2 Within-person results

#### 4.2.1 The longitudinal associations between relative deprivation and prosocial behavior

This study found that prosocial behavior was significantly negatively associated with subsequent relative deprivation at the within-person level. This result supports Hypothesis 2 and extends previous cross-sectional research (20, 22, 23). College students are in a period of unstable emotional development, and their exposure to hierarchical evaluation systems and peer-oriented campus environments may render them particularly prone to social comparison (11). Increasing competition for further education and employment can heighten the frequency of such comparisons, which may easily trigger feelings of relative deprivation (5). Research has revealed that engaging in prosocial behaviors can lead to positive emotions. The broaden-and-build theory suggests that positive emotions can broaden college students' cognitive scope, and thereby reduce negative emotions, such as anger and dissatisfaction, induced by relative deprivation (19). Consequently, individuals become more accepting of interpersonal differences and engage less in excessive comparisons of gains and losses, ultimately associated with lower levels of relative deprivation (12). Moreover, engaging in prosocial behavior can effectively satisfy college students' basic psychological needs (20). College students are in a critical period of identity development, and the self-determination theory suggests that satisfying these basic psychological needs can help build a stable intrinsic value system (21, 65). Such a system can reduce their reliance on social comparisons to verify self-worth (12). Since relative deprivation is often shaped by evaluating self-worth through social comparisons, meeting the basic psychological needs via prosocial behavior may help students shift their source of self-worth away from social comparisons, and, in turn, be associated with weaker relative deprivation (12, 20, 22). This provides indirect support for our hypotheses. Therefore, we can conclude that prosocial behavior is predictive of subsequent changes in relative deprivation.

However, we found that relative deprivation did not have a direct predictive association with subsequent prosocial behavior, which does not support Hypothesis 1. We infer that this may be because relative deprivation is understood as a factor that indirectly influences prosocial behavior. Research has suggested that relative deprivation is more closely linked to internal distress, such as depression, anxiety, and loneliness, rather than directly associated with lower levels of external prosocial behavior (66, 67). From the perspective of the cognitive-behavioral theory, cognition is a primary determinant of behavior (68). Consequently, prosocial behavior is more likely to be influenced by cognitive factors, such as moral standards and personal values (34, 35). A longitudinal study conducted among college students found that relative deprivation did not directly predict cyberbullying behavior across time. Instead, their longitudinal association was mediated by revenge (13). This finding indirectly supports our perspective. Thus, relative deprivation appeared to be indirectly associated with prosocial behavior by shaping individuals' cognition.

Collectively, this suggests that prosocial behavior can directly predict subsequent relative deprivation, whereas relative deprivation does not directly predict prosocial behavior. Furthermore, the reverse path (prosocial behavior to relative deprivation) is stronger, and the underlying reasons can be further explained. The self-perception theory proposes that individuals adjust their internal cognitive judgments and emotional evaluations by observing their own behavioral performances (69). Prosocial behavior, an explicit and observable form of social interaction, is associated with immediate social feedback, such as recognition from others and a sense of competence, which is linked to subsequent fluctuations in individuals' self-evaluations and reference standards for social comparison (20, 21). In contrast, the association between relative deprivation and prosocial behavior may involve more intricate internal psychological transformation processes (13). Prosocial behavior is generally constrained by social norms and personal moral principles (34, 35), whereas relative deprivation reflects an internal cognitive and emotional state (12). Short-term fluctuations in relative deprivation may not be sufficient to be directly linked to changes in behavioral patterns. Importantly, our results indicate a significant positive association between relative deprivation and subsequent psychological entitlement; however, no direct association was observed between relative deprivation and prosocial behavior. This further suggests that the association between relative deprivation and prosocial behavior is linked to the cognitive mechanism of psychological entitlement at the within-person level across specific time points. Accordingly, in our study, the reverse association from prosocial behavior to relative deprivation is stronger.

#### 4.2.2 The mediating role of psychological entitlement

We found that psychological entitlement at Time 2 mediated the association between relative deprivation at Time 1 and prosocial behavior at Time 3, supporting Hypothesis 3. These within-person mediation effects reflect dynamic within-person changes over time, controlled for time-invariant between-person traits. Specifically, deviations from one's own typical level of relative deprivation at T1 may be associated with corresponding changes in psychological entitlement at T2, which may be linked to subsequent changes in prosocial behavior at T3. Previous studies have mainly adopted cross-sectional designs to investigate the negative association between relative deprivation and prosocial behavior, and focused exclusively on inter-individual trait differences (7–9). Such designs limit understanding of how perceptions of unfairness arising from relative deprivation may be linked to changes in prosocial behavior over time and leave the underlying cognitive mechanisms unclear. Our longitudinal findings indicate that when an individual's sense of relative deprivation at a particular moment surpasses their usual level, it may be accompanied by stronger unfairness perceptions, greater focus on personal deservingness, and compensatory demands (12, 26). These patterns may relate to lower empathy and moral concern (24, 32), which can relate to weaker prosocial tendencies (35). These results imply that the association between relative deprivation and prosocial behavior may not be direct; rather, it appears to be mediated by egocentric cognitions, such as psychological entitlement. This finding advances existing research in two ways. First, it provides longitudinal support for the equity theory from a within-person dynamic perspective (26). At the within-person level, changes in fairness perceptions arising from relative deprivation do not appear to drive behavioral changes directly. Instead, such changes may be associated with gradually lower levels of prosocial behavior, alongside the sustained role of psychological entitlement. Second, prior research has largely explained this association by focusing on negative emotions or resource-protection motives, often at the between-person level (8, 16, 29). However, our study adopts a social cognitive processing perspective (31). It illuminates a potential indirect mechanism linking relative deprivation and prosocial behavior at the within-person longitudinal level (30). It suggests that psychological entitlement may serve an important function in the dynamic processes through which relative deprivation is related to prosocial behavior.

Our study also found that psychological entitlement at Time 2 mediated the association between prosocial behavior at Time 1 and relative deprivation at Time 3, supporting Hypothesis 4. These within-person mediation effects reflect dynamic within-person changes over time, controlled for time-invariant between-person traits. That is, within-person deviations from one's own typical level of prosocial behavior at T1 may be associated with corresponding changes in psychological entitlement at T2, which may be linked to subsequent changes in relative deprivation at T3. Previous studies have primarily used cross-sectional designs to examine the association between prosocial behavior and relative deprivation, and focused only on between-person trait differences (37, 41). This approach made it difficult to understand how cognitive changes resulting from prosocial behavior may relate to relative deprivation at the within-person level over time; furthermore, the mechanisms underlying this link remain unclarified. Our longitudinal findings indicate that when an individual's sense of prosocial behavior at a particular moment surpasses their usual level, it enhances their social connectedness and sense of self-worth and weakens egocentric cognition, which could be linked to lower psychological entitlement (24, 36, 37), and may further predict alleviated relative deprivation (30, 42). This suggests that the longitudinal relationship between these two is also mediated by perceived psychological entitlement. These findings advance existing research in two ways. First, it offers new empirical support for the dynamic interaction theory. Prosocial behavior may be associated with gradually weaker relative deprivation at the within-person level, potentially through the role of psychological entitlement; this illustrates a dynamic process in which behavioral responses may be linked to shifts in psychological states via cognitive mediation. Second, this study provides longitudinal empirical evidence to supplement Adler's theory of social interest (36, 37). It clarifies the inherent logical pathway in which prosocial behavior may correlate with psychological entitlement through variations in social bonds and egocentric cognition, accompanied by subsequent fluctuations in relative deprivation experiences.

Notably, the within-person cross-lagged and indirect effects were modest in magnitude, which may not be uncommon in RI-CLPM (70, 71). Such within-person effects are generally weaker, as the model separates stable between-person traits from time-varying within-person fluctuations. This statistical separation naturally reduces the magnitude of the longitudinal path coefficients (49, 72). Furthermore, relative deprivation, psychological entitlement, and prosocial behavior are mutually associated and may also be shaped by short-term situational changes, such as transient events in school climate and family environments (4, 14, 24). We primarily focus on the longitudinal mediating pathways among the three focal variables and do not account for all relevant short-term situational factors. These excluded external transient influences may have contributed to some variability and weakened the predictive associations observed. This may help explain the relatively modest within-person effect sizes found in this study.

### 4.3 Implications, limitations, and future directions

This study has theoretical and practical implications. Theoretically, it captures the dynamic temporal associations between relative deprivation, psychological entitlement, and prosocial behavior. Our results extend existing literature and provide empirical support for relevant theoretical frameworks. Moreover, we found that relative deprivation does not predict prosocial behavior at the within-person level, which enriches understanding of the association between relative deprivation and prosocial behavior. These results indicate that the direct negative association between relative deprivation and prosocial behavior is primarily manifested at the trait level across individuals, rather than at the level of intra-individual situational fluctuations. Simultaneously, regarding short-term fluctuations within individuals, the findings suggest that the association between relative deprivation and prosocial behavior requires the mediating role of cognitive factors, such as a sense of psychological entitlement. This underscores the need to distinguish within-person variability from between-person differences and validates the unique value of the longitudinal design. Additionally, although the effects estimated by the RI-CLPM model at the within-person level were generally modest, these stable, small effect sizes may not be considered insignificant; even small but consistent within-person effects may have important theoretical implications and help reveal the dynamic nature of psychological processes (73). This study also offers some practical insights. Higher education institutions can appropriately focus on fostering students' prosocial behaviors by providing volunteer services, peer support, and public service initiatives to create additional opportunities for students to participate in various social service activities. For student groups experiencing strong relative deprivation, such as those facing financial hardship or chronic academic stress, providing appropriate psychological support may encourage greater participation in prosocial activities. Simultaneously, psychological entitlement is a psychological characteristic closely linked to an individual's life experiences and subject to dynamic change, rather than an innate, fixed, or permanently stable personality trait (24). Advocating egalitarian values, enhancing individual empathy, and correcting irrational cognitive biases may be negatively correlated with students' tendencies toward psychological entitlement to some extent. Such approaches may be associated with lower relative deprivation and higher levels of prosocial behavior. Furthermore, although the cross-lagged paths and indirect effects yielded modest magnitudes at the within-person level, such small effect sizes often reflect slow and stable long-term psychological processes (73). Such linkages may not be salient in the short run, whereas sustained, targeted supportive resources may correlate with cumulative positive changes in individuals' psychological functioning and behavioral development over an extended period (74). Accordingly, the longitudinal associations between the variables can be better interpreted from a long-term developmental perspective. Consistent and stable supportive environments may relate to fewer negative emotional experiences, better cognitive adjustment, and more stable prosocial behavioral tendencies.

Nevertheless, this study has certain limitations. First, all focal variables were evaluated using multi-wave self-report questionnaires, which may be influenced by social desirability bias and self-perception bias. While Harman's single-factor test and one-factor confirmatory factor analysis (CFA) were used to assess common method bias, these *post-hoc* analyses are limited in their ability to eliminate it completely. Notably, prosocial behavior is particularly prone to social desirability bias, may lead adolescents to overestimate their prosocial tendencies. Therefore, future studies should consider using multi-method assessments, such as peer nominations, teacher evaluations, and behavioral observations, to reduce common-source bias and enhance measurement reliability. Second, the sample was selected from a single Chinese University using convenience sampling, which may limit the generalizability of the findings. Future research should consider including samples from multiple universities to enhance the external validity and generalizability of the results. Third, the magnitudes of the longitudinal mediating and path effects were relatively small at the within-level. Although such modest effect sizes may not be uncommon in within-person longitudinal research, subsequent studies can adopt longer time intervals and further intensive repeated measurements to capture more pronounced dynamic associations and verify the robustness of the findings. Finally, we only examined the mediating role of psychological entitlement. Exploring other potential moderating factors, such as academic performance, campus climate, and family circumstances, would be valuable to deepen the longitudinal associations between relative deprivation, psychological entitlement, and prosocial behavior among college students at both the within-and between-person levels.

## 5 Conclusion

This study analyzed three waves of longitudinal data collected from college students. It employed the RI-CLPM to explore the longitudinal relationship between relative deprivation and prosocial behavior, and the mediating role of psychological entitlement. We found that relative deprivation was significantly positively associated with psychological entitlement at the between-person level. Furthermore, both were significantly negatively associated with prosocial behavior; at the within-person level, prosocial behavior negatively predicted longitudinal relative deprivation, whereas relative deprivation did not predict longitudinal prosocial behavior. Furthermore, psychological entitlement mediated the longitudinal relationship between relative deprivation and prosocial behavior.

## Data Availability

The raw data supporting the conclusions of this article will be made available by the authors, without undue reservation.
