# Supplementary material for: The mediating role of psychological entitlement in the relationship between relative deprivation and prosocial behavior among college students: a random intercept cross-lagged panel model
Source: Front Public Health. 2026 Jun 3;14:1821940. doi: 10.3389/fpubh.2026.1821940 (PMC13271942; doi:10.3389/fpubh.2026.1821940)
Supplement: Supplementary file 1 [file Data_Sheet_1.pdf]

Supplementary Material

Supplementary A

Table 1 RI-CLPM estimates (unstandardized)

	Est	S.E.	*P*	95%CI
Covariate				
Gender→ relative deprivation	-0.023	0.037	0.529	[-0.083,0.037]
Gender→ psychological entitlement	-0.009	0.044	0.843	[-0.082,0.064]
Gender→ prosocial behavior	0.005	0.021	0.798	[-0.029,0.040]
Between-person effects				
relative deprivation with psychological entitlement	0.153	0.054	0.005	[0.064,0.242]
relative deprivation with prosocial behavior	-0.080	0.022	<0.001	[-0.117,-0.044]
psychological entitlement with prosocial behavior	-0.082	0.030	0.006	[-0.131,-0.033]
Within-person effects				
Autoregressive paths				
relative deprivation$_{T1}$→relative deprivation$_{T2}$	0.208	0.042	<0.001	[0.139,0.276]
relative deprivation$_{T2}$→relative deprivation$_{T3}$	0.208	0.042	<0.001	[0.139,0.276]
psychological entitlement$_{T1}$→psychological entitlement$_{T2}$	0.205	0.049	<0.001	[0.125,0.286]
psychological entitlement$_{T2}$→psychological entitlement$_{T3}$	0.205	0.049	<0.001	[0.125,0.286]
prosocial behavior$_{T1}$→prosocial behavior$_{T2}$	0.127	0.036	<0.001	[0.068,0.187]
prosocial behavior$_{T2}$→prosocial behavior$_{T3}$	0.127	0.036	<0.001	[0.068,0.187]
Cross-lagged paths				
relative deprivation$_{T1}$→psychological entitlement$_{T2}$	0.214	0.039	<0.001	[0.150,0.279]
relative deprivation$_{T1}$→prosocial behavior$_{T2}$	-0.010	0.018	0.588	[-0.040,0.020]
relative deprivation$_{T2}$→psychological entitlement$_{T3}$	0.214	0.039	<0.001	[0.150,0.279]
relative deprivation$_{T2}$→prosocial behavior$_{T3}$	-0.010	0.018	0.588	[-0.040,0.020]
psychological entitlement$_{T1}$→relative deprivation$_{T2}$	0.095	0.029	0.001	[0.048,0.142]
psychological entitlement$_{T1}$→prosocial behavior$_{T2}$	-0.133	0.016	<0.001	[-0.160,-0.106]
psychological entitlement$_{T2}$→relative deprivation$_{T3}$	0.095	0.029	0.001	[0.048,0.142]
psychological entitlement$_{T2}$→prosocial behavior$_{T3}$	-0.133	0.016	<0.001	[-0.160,-0.106]
prosocial behavior$_{T1}$→relative deprivation$_{T2}$	-0.216	0.050	<0.001	[-0.299,-0.134]
prosocial behavior$_{T1}$→psychological entitlement$_{T2}$	-0.306	0.057	<0.001	[-0.400,-0.212]
prosocial behavior$_{T2}$→relative deprivation$_{T3}$	-0.216	0.050	<0.001	[-0.299,-0.134]

prosocial behavior$_{T2}$→psychological entitlement$_{T3}$	-0.306	0.057	<0.001	[-0.400,-0.212]
Residuals				
relative deprivation$_{T1}$ with psychological entitlement$_{T1}$	0.172	0.058	0.003	[0.077,0.268]
relative deprivation$_{T2}$ with psychological entitlement$_{T2}$	0.084	0.031	0.008	[0.032,0.135]
relative deprivation$_{T3}$ with psychological entitlement$_{T3}$	0.192	0.032	<0.001	[0.138,0.245]
relative deprivation$_{T1}$ with prosocial behavior$_{T1}$	-0.108	0.027	<0.001	[-0.153,-0.063]
relative deprivation$_{T2}$ with prosocial behavior$_{T2}$	-0.064	0.016	<0.001	[-0.090,-0.038]
relative deprivation$_{T3}$ with prosocial behavior$_{T3}$	-0.070	0.015	<0.001	[-0.095,-0.046]
psychological entitlement$_{T1}$ with prosocial behavior$_{T1}$	-0.183	0.033	<0.001	[-0.236,-0.129]
psychological entitlement$_{T2}$ with prosocial behavior$_{T2}$	-0.085	0.018	<0.001	[-0.114,-0.055]
psychological entitlement$_{T3}$ with prosocial behavior$_{T3}$	-0.063	0.018	0.001	[-0.092,-0.033]

Supplementary B

Mplus syntax (M4, autoregressive and cross-lagged paths fixed to be time-invariant)

```
DATA:
    FILE IS D:\Users\46593\Desktop\RICLPM.dat;
    VARIABLE:
    NAMES ARE SEX T1_X  T1_M  T1_Y T2_X T2_M T2_Y  T3_X  T3_M  T3_Y ;
    USEVARIABLE ARE SEX T1_X  T1_M T1_Y T2_X T2_M T2_Y T3_X T3_M T3_Y;
    ANALYSIS:
      ESTIMATOR = ML;
      BOOTSTRAP=2000;
      MODEL = NOCOV;
    MODEL:
      BVX BY T1_X@1 T2_X@1 T3_X@1;
      BVM BY T1_M@1 T2_M@1 T3_M@1;
      BVY BY T1_Y@1 T2_Y@1 T3_Y@1;
      BVX WITH BVM BVY;
      BVM WITH BVY;
      WT1_X BY T1_X@1;
      WT2_X BY T2_X@1;
```

```
WT3_X BY T3_X@1;
WT1_M BY T1_M@1;
WT2_M BY T2_M@1;
WT3_M BY T3_M@1;
WT1_Y BY T1_Y@1;
WT2_Y BY T2_Y@1;
WT3_Y BY T3_Y@1;
T1_X-T3_Y @ 0;
WT2_M ON WT1_M(d);
WT2_Y ON WT1_Y(e);
WT2_X ON WT1_X(f);
WT3_M ON WT2_M(d);
WT3_Y ON WT2_Y(e);
WT3_X ON WT2_X(f);
WT2_X ON WT1_M(g);
WT2_X ON WT1_Y(h);
WT2_M ON WT1_X(i);
WT2_M ON WT1_Y(j);
WT2_Y ON WT1_X(k);
WT2_Y ON WT1_M(l);
WT3_X ON WT2_M(g);
WT3_X ON WT2_Y(h);
WT3_M ON WT2_X(i);
WT3_M ON WT2_Y(j);
WT3_Y ON WT2_X(k);
WT3_Y ON WT2_M(l);
WT1_X WITH WT1_M WT1_Y;
WT1_M WITH WT1_Y;
WT2_X WITH WT2_M WT2_Y;
WT2_M WITH WT2_Y;
```

```
WT3_X WITH WT3_M WT3_Y;
WT3_M WITH WT3_Y;
BVX BVM BVY ON SEX;
MODEL INDIRECT:
WT3_Y IND WT2_M WT1_X;
WT3_X IND WT2_M WT1_Y;
OUTPUT:TECH1 STDYX SAMPSTAT CINTERVAL;
```
